# How to choose initial treatment in multiple sclerosis patients: a case-based approach

**DOI:** 10.1590/0004-282X-ANP-2022-S128

**Published:** 2022-08-12

**Authors:** Samira Luisa Pereira Apóstolos, Mateus Boaventura, Natalia Trombini Mendes, Larissa Silva Teixeira, Igor Gusmão Campana

**Affiliations:** 1 Universidade de São Paulo, Faculdade de Medicina, Hospital das Clínicas, Departamento de Neurologia, São Paulo SP, Brazil.

**Keywords:** Multiple Sclerosis, Therapeutics, Esclerose Múltipla, Terapêutica

## Abstract

**Background:**

Immunotherapy dramatically changed the natural history of multiple sclerosis (MS), which was classically associated with severe disability. Treatment strategies advocate that early control of disease activity is crucial to avoid progressive disability, and the use of high efficacy drugs may be beneficial, but safety is a concern. Choosing the disease-modifying therapy is challenging in clinical practice and should be further discussed.

**Objective:**

To discuss the state of art of selecting the initial therapy for relapsing MS patients.

**Methods:**

We used a case-based approach followed by clinical discussion, exploring therapeutic options in different MS settings.

**Results:**

We presented clinical cases profile compatible with the use of MS therapies, classified into moderate and high efficacy. In the moderate efficacy group, we discussed interferons, glatiramer acetate, teriflunomide and dimethyl fumarate, while in the high efficacy group we discussed fingolimod, cladribine, natalizumab, ocrelizumab, alemtuzumab and ofatumumab.

**Conclusion:**

Advances in MS treatment are remarkable. Strong evidence supports the use of early high efficacy therapy. However, biomarkers, clinical and radiologic prognostic factors, as well as patients' individual issues, should be valued and considered for a personalized treatment decision.

## RESUMO

**Antecedentes:**

A imunoterapia mudou drasticamente a história natural da esclerose múltipla (EM), doença esta que era classicamente associada a grandes incapacidades. Sabe-se hoje que o controle precoce da atividade de doença é crucial para evitar incapacidade progressiva, e o uso de terapias de alta eficácia pode ser benéfico. Apesar disso, a segurança ainda é uma preocupação dos pacientes e médicos. A escolha da terapia modificadora da doença é um desafio na prática clínica e suas particularidades devem ser mais discutidas.

**Objetivo:**

Discutir o estado da arte da seleção da terapia inicial para pacientes com EM remitente recorrente.

**Métodos:**

Utilizamos uma abordagem baseada em casos clínicos, com discussão das diversas opções terapêuticas em diferentes contextos de EM.

**Resultados:**

Foram apresentados casos clínicos compatíveis com o uso das principais terapias para EM, divididas em moderada e alta eficácia. No grupo de moderada eficácia discutimos sobre os interferons, acetato de glatirâmer, teriflunomida e fumarato de dimetila enquanto que no de alta eficácia falamos sobre fingolimode, cladribina, natalizumabe, ocrelizumabe, alentuzumabe e ofatumumabe.

**Conclusão:**

Os avanços no tratamento da EM são notáveis. Fortes evidências suportam que o uso de terapia de alta eficácia de forma precoce possa ser benéfica. No entanto, biomarcadores, fatores prognósticos clínicos e radiológicos, bem como questões individuais dos pacientes, devem ser valorizados e considerados para uma decisão de tratamento personalizado.

## INTRODUCTION

Multiple sclerosis (MS) is a chronic, autoimmune, inflammatory, demyelinating and neurodegenerative disease of the Central Nervous System^1^. Clinical, neuropathological, imaging, and biomarker data suggest a continuous destructive process across all clinical stages of MS^2^. It represents the main cause of neurological disability in young adults, leading to social and personal burden and is associated with around five years reduction in life expectancy^3^ ^,^ ^4^.

The clinical classification of MS comprises: (1) a latent period (prodrome), followed probably by (2) radiological and/or clinical activity - respectively, radiologically isolated syndrome (RIS) or Clinically isolated syndrome (CIS) - and trailed by (3) clinically definite MS, both relapsing and progressive forms, each of them being able to evolve with and without activity. RIS is defined as the presence of MS typical magnetic resonance imaging (MRI) brain lesions in an asymptomatic person^5^ ^,^ ^6^ and treatment approach to RIS will not be discussed here. CIS is defined as a monofocal or multifocal first clinical event suggestive of relapsing MS (RMS) in a person not meeting complete diagnosis criteria. It stands as a risk for transition to MS over time, and usually is treated as RMS^6^. MS diagnosis criteria include a clinical-laboratorial evaluation showing dissemination on space and time - usually relapse, new MRI lesions or presence of oligoclonal bands (OCB)^5^ ^,^ ^6^. Acute neurological deterioration - or relapse associated worsening (RAW) - and progression independent of relapse (PIRA), may occur in RMS course [2]. Progressive MS, both primary progressive or secondary progressive, recently has a growing range of therapeutic options, but is beyond the scope of this paper. RMS is the most common disease type and initial presentation.

**Figure 1.**
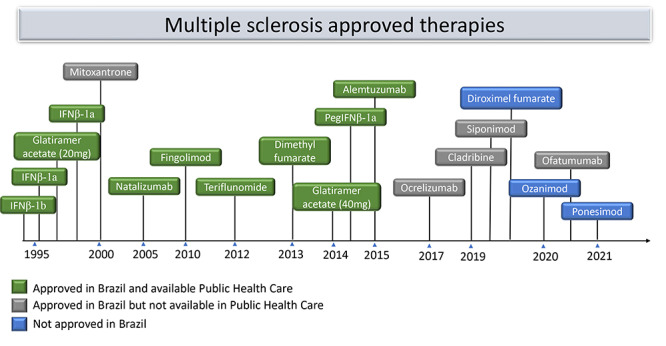
Landscape of approved therapy - 2022.

IFN: Interferon; Peg: Pegylated.

Early treatment avoids conversion to clinically defined MS in CIS and progressive disability in all RMS patients^7^ ^,^ ^8^. Treatment aims to prevent disease activity and progression. Disease Activity (DA) is determined based on (1) clinical relapses and (2) MRI activity - contrast-enhancing lesions (CEL), new or enlarged T2-lesions. Progression is determined by adding disability on regular evaluation. This echoes the concept of no evidence of disease activity (NEDA), defined by absence of relapses, MRI activity and disease progression^9^. The development of disease modifying therapy (DMT) with diverse efficacy and safety profile provides wide therapeutic possibilities and choosing the initial drug to manage MS patients becomes challenging, mainly following the COVID-19 pandemic^10^ (Figure 1). The most common therapeutic approach has been divided as escalation versus induction therapy^6^.

According to the escalation approach, lower- to moderate-efficacy therapies with a known and relatively safe risk profile are selected for initial treatment (first-line) and switched to a second- or third-line drug according to disease activity. The rationale behind the escalation strategy is that patients at the earlier disease stage may respond optimally to safer and lower efficacy DMT. The escalation approach classifies DMT into “lines” of treatment and supports therapeutic guidelines for MS in several regulatory agencies, including Europe and the Brazilian Health System^11^ ^.^ A second line is controversial. Fingolimod, for example, is used as second-line therapy in Europe, while it's a first-line therapy in the USA^12^. The concept of in-line therapy is not recommended and It has been replaced by a strategy of classifying DMT according to efficacy. Taking into account the average annualized relapse range (ARR) reduction, found in the original pivotal studies, two broad classes are recognized: (1) between 30 and 50% - Moderate Efficacy Therapy (MET) or (2) substantially more than 50% - High Efficacy Therapy (HET)^13^. By adding to this concept, the data from real world comparative observational studies, DMT classified as HET include alemtuzumab, natalizumab, ocrelizumab, ofatumumab, cladribine, fingolimod, while MET includes dimethyl fumarate, glatiramer acetate, IFN-β preparations and teriflunomide^13^.

On the other hand, induction therapy is based on selecting a HET at the time of diagnosis, in order to achieve early disease control. The rationale behind the induction strategy relies on “resetting” the immune system, using high efficacy and high immunosuppressive DMT, ideally lasting as short a time as possible, to minimize the risk of malignant neoplasms and opportunistic infections^14^. In clinical practice, the use of the term “induction therapy” may be confusing and has generated some resistance from clinicians/patients worried about risk aversion. In addition, instead of use the term “induction therapy”, the term high efficacy maintenance therapy or high efficacy induction therapy has been preferred.. Meanwhile, the escalation-based treatment approach has no lasting clinical benefits and may curtail the patient's so-called “window of opportunity” of better benefit with HET, since all DMTs have better efficacy in the earlier stages of MS^14^. In this article, we shall use the concept of efficacy therapy.

Over the last three decades, immunotherapy dramatically modified the natural history of MS and heralded the era of treatment in neurodegenerative diseases^15^. We discuss the state-of-art of how to start treatment in patients with relapsing MS using a case-based approach in two scenarios: 1 - When to use a moderate efficacy therapy (MET) and 2 - When to use a High Efficacy therapy (HET). 

## CASE PRESENTATION AND THERAPY DISCUSSION - WHEN TO CHOOSE MODERATE EFFICACY THERAPY

### Case description 01

A 20-year-old female with a history of an isolated episode of a mild optic neuritis two years ago with complete recovery without treatment, comes for a routine neurologic evaluation. Her brain and spinal MRI at the time of the relapse were unremarkable, cerebrospinal fluid (CSF) was normal and OCB were absent, anti-AQP4 and anti-MOG antibodies were negative. New brain MRI showed three T2-lesions typical for the diagnosis of MS. No CEL or infratentorial lesions were found on MRI. Her parents and the patient were concerned about infection risks. 

*Interferons*

Interferon-beta (IFNβ) is among the first DMT proven effective in the treatment of CIS and RMS and is approved for the treatment of both adults and children^16^. Its mechanisms of action are complex and include avoiding leukocyte migration across the blood-brain barrier, induction of regulatory T cells and inhibition of autoreactive T cells^17^.

IFNβ reduction in the ARR ranges from 27% to 36% compared to placebo^18^ ^-^ ^20^ and MRI activity (new or active lesions) reduction reaches about 60%^21^ in the main randomized controlled trials.

There are different formulations of IFNβ approved for the treatment of MS, which mainly differ by route and frequency of administration: subcutaneous IFNβ-1b every other day, subcutaneous three times a week or intramuscular weekly IFNβ-1a and subcutaneous every two weeks pegylated IFNβ-1a; their characteristics are summarized in Table 1. 

**Table 1.** Summary characteristics of disease modifying therapies for relapsing multiple sclerosis.

	Administration route and dosage	ARR reduction†	Rates of sustained NEDA 3 (time)
Moderate efficacy			
Interferon Beta	Injectable (IM or SC)	28-34%^18-20^	
IFNβ-1a	IM - 30ug weekly		14% in DECIDE study (96wk)
	SC - 22ug or 44ug 3x/week		**
IFNβ-1b	SC - 250ug every other day		27% in pooled data from OPERA I and OPERA II studies (96wk)
Peg IFNβ-1a	SC- 125ug every 2 weeks		37% in ADVANCE study (2yr)
Glatiramer Acetate	SC - 40mg 3x/week or 20mg once daily	29%^24^	**
Dimethyl fumarate	Oral - 240mg twice daily	44-53%^35,36^	27% in pooled data from CONFIRM and DEFINE studies (2yr)
Teriflunomide	Oral - 7mg or 14mg once daily	31-36%^28,29^	23% in TEMSO study
High efficacy			
Fingolimod	Oral - 0.5mg once daily	51-55%^42,43^	31% in pooled data from FREEDOMS and FREEDOMS II studies (2yr)
Cladribine	Oral - 1.75mg/kg/year taken in 2 weeks. Repeat course after 12 months	58%^50^	30% in CLARITY Extension study (2yr)
Ofatumumab	SC - 20mg monthly after initial load	50-60%^66^	42% in pooled data from ASCLEPIOS I and II trials (2yr)
Natalizumab	IV - 300mg every 4 weeks	54-68%^51,52^	37% in AFFIRM study (2yr)
Alemtuzumab	IV - 2 cycle, 12 months apart: 12mg/day for 5 days and 12mg/day for 3 days	55%^57^	61% in CARE MS I Extension Study (3yr)
Ocrelizumab	IV - 600mg every 6 months	46-47%^63^	48% in pooled data from OPERA I and OPERA II studies (96wk)

ARR: Annualized Relapse Rate; INFB - Beta interferonas, Peg INF - beta interferona peglada.. †Data from pivotal trials compared to control group (either placebo or active group).

Common adverse effects include headache, injection site reaction and flu-like symptoms. The treatment should also be avoided in patients with psychiatric disorders, since depression and suicidal risks are increased through IFNβ therapy. Asymptomatic elevation of liver enzymes is common. Severe adverse reactions are rare and include liver disease, thrombotic microangiopathy, hemolytic anemia, allergic reactions, congestive heart failure and seizures^17^. Before starting IFN therapy complete blood count (CBC) and liver enzymes are necessary and patients should be monitored with these same exams every six months. 

Although the adverse reactions and lower efficacy may limit the use of IFNβ nowadays, it is considered safer than some higher efficacy DMT, particularly concerning the risk for infections^22^ and for specific groups of patients, such as children and pregnant women.

In this case, good prognostic factors, mild disease onset and shared-decision about infection and risk aversion conducted towards IFN therapy.

### Case description 02

A 25-year-old female presented hypoesthesia on the right side of her face, lasting 10 days, with complete and spontaneous recovery. Neurological examination was normal. She expressed a desire to get pregnant soon. Brain MRI showed typical lesions with low lesion burden, compatible with the diagnosis of MS. Spine MRI was unremarkable. OCB was absent in CSF. She came for a second opinion about treatment since Cladribine has been recommended before getting pregnant. She was concerned about safety, but wanted to treat the disease as soon as possible. 

*Glatiramer acetate*

Glatiramer acetate (GA) is an injectable medication approved for CIS and MS. Its structure is similar to myelin basic protein, which means that there is a competition between glatiramer acetate and the various myelin antigens for their presentation to T lymphocytes^23^.

Glatiramer reduces the ARR by around 30% when compared to placebo in pivotal studies^24^. Recently, the benefit of a more convenient dose, with a 34% reduction in AAR, 44% in new CEL and 34.7% for new T2 lesions has been found^25^. GA is used subcutaneously, with two posological options: 20mg daily; or 40mg three times a week. Due to convenience, the dose of 40mg is the most used^24^ ^,^ ^25^.

Side effects of GA include local injection site reactions and, less commonly, systemic manifestations after administration, such as palpitation, dyspnea, and anxiety^24^. There are rare reports of serious adverse events, mainly related to hepatotoxicity^26^. There is no need for any exams to initiate or monitor the use of GA. This is a medication with an excellent safety profile, but due to its low efficacy its use has become restricted. GA is mostly used for children under 18 years of age, women with a desire for pregnancy or patients who want safety over efficacy. 

Due to the patient's desire and risk aversion, mild relapse and low lesion load, we discussed and decided on the use of GA.

### Case description 03

A 48 year-old female is admitted for a neurologic evaluation after presenting an episode of trigeminal neuralgia with complete spontaneous recovery. Her brain MRI showed a total of eight lesions distributed in the periventricular, cortical and infratentorial areas. Spinal MRI did not show any lesions. She had two children and had a definitive contraception a few years ago. The patient desired to start an oral medication and was concerned about infection risk. 

*Teriflunomide*

Teriflunomide, an oral once-a-day tablet, is approved for CIS and RMS treatment. It is a reversible inhibitor of dihydroorotate dehydrogenase, a mitochondrial enzyme involved in pyrimidine synthesis and DNA replication of highly proliferating cells. It causes a cytostatic effect on proliferating T and B lymphocytes without affecting resting lymphocytes^27^.

The randomized controlled trials TEMSO^28^ and TOWER^29^ evaluated the efficacy of oral teriflunomide versus placebo, showing that 14mg daily reduced the ARR by 31% and 36%, respectively. MRI outcomes were assessed by the TEMSO study, which revealed a reduction of 67% of total lesion volume compared to placebo.

Common adverse effects include headache, nausea, diarrhea, hair thinning and hepatic enzymes increase (mild and transient in most cases). Patients treated with teriflunomide don’t appear to have higher risk of infections and the frequency of serious adverse events was similar across treatment and placebo groups^30^.

Administration of teriflunomide during pregnancy may be teratogenic in animal studies. Thus, its use in women of reproductive potential not using effective contraception is not recommended. For patients who accidentally become pregnant or want to start a family during treatment, it is recommended to accelerate the elimination of teriflunomide with cholestyramine or activated charcoal, as it takes an average of eight months for the natural elimination of the drug to occur^31^.

Before the initiation of teriflunomide, CBC, hepatic enzymes and TSH should be obtained. Pregnancy and latent tuberculosis must be excluded. Monitoring includes CBC and hepatic enzymes monthly until six months of treatment and every six months afterwards^31^. 

Treatment with teriflunomide has a convenient dosage regimen, a good safety profile and it should be considered in patients with low disease activity and without childbearing potential. 

### Case description 04

A 22-year-old female presented with unilateral optic neuritis 6 months ago and had a full recovery after pulse therapy with methylprednisolone. Current EDSS is 1.0 (Afferent Pupillary Defect) and MRI showed juxtacortical and periventricular lesions with no CEL, a normal spine MRI and no OCBs was found in CSF. Due to a non-aggressive MS course and the delay for starting DMT until then, immediate initiation of Dimethyl fumarate (DMF) was chosen.

*Dimethyl fumarate*

DMF, a twice-a-day oral agent, is approved to treat RMS. It reduces the number of circulating T cells, particularly CD8+ T cells, thereby suppressing immune responses^32^. Its key molecular mechanism may be due to a general downregulation of glycolysis, especially in cells with high metabolic turnover, mainly affecting effector and memory T cells^33^. Besides, it is involved in the activation of nuclear factor (erythroid-derived 2)-type 2 (Nrf2) transcription pathway, and it has been shown to upregulate Nrf2 dependent antioxidant genes in the patients^34^.

Two pivotal studies (CONFIRM [35] and DEFINE^35^) showed an ARR relative reduction of 44% and 53% when DMF was compared with placebo, respectively. CONFIRM data presented reduction rates of new T2-lesions and CEL of 71% and 74%, respectively^36^.

Fumarate use should begin with an oral dose of 120 mg twice daily for seven days, followed by a continued dose of 240 mg twice daily^37^.

Very commonly reported adverse effects include flushing (up to 35% of patients) and Gastrointestinal (GI) disorders (up 44%), both presented mainly in the first month of use and constituting the main reasons for treatment discontinuation^36^ ^,^ ^38^ Strategies to minimize adverse effects include: nutritional counseling, use of proton-pump inhibitors and symptomatic drugs for dyspepsia, and use of aspirin 30 minutes before taking the drug, to avoid flushing^39^. Recently, a new once-a-day fumarate formulation (viroximel fumarate) reduces the risk of GI symptoms and provides similar therapeutic benefits of DMF. In addition, adverse events such as liver disorders with elevated transaminases, renal dysfunction with proteinuria or hematuria, lymphopenia or even serious infections may occur^38^. Because of this, routine periodic monitoring with urea, creatinine, urinalysis, transaminases, bilirubins and blood count is recommended, as it is at the initial assessment before starting the medication. Reports of Progressive Multifocal Leukoencephalopathy (PML) were rare and associated with patients with severe persistent lymphopenia^40^.

Lastly, a real-life study showed that the following characteristics are predictors of good response to DMF: a - younger patients at diagnosis, b - use of DMF as first line of treatment - avoid using as a de-escalation strategy - c - shorter disease duration and d - lower EDSS at the beginning of therapy^41^.

Fumarate was chosen for this patient considering a non-aggressive early diagnosed disease with the possibility of immediate initiation of a disease-modifying treatment, without the need for extensive efforts for monitoring or preparation.

## CASE PRESENTATION AND THERAPY DISCUSSION - WHEN TO CHOOSE HIGH EFFICACY THERAPY

### Case description 05

A 23-year-old male patient presented with low visual acuity, with partial recovery after methylprednisolone. On neurological examination he presented a visual acuity of 20/50 and a relative pupillary defect in the right-eye. Brain MRI showed > 10 T2 lesions (periventricular, juxtacortical) without CEL. Spinal MRI was unremarkable. OCB was positive in CSF. He had a positive anti-JCV (John Cunningham Virus) antibody and varicella zoster IgG positive. Electrocardiogram was normal. He desired to start on an oral medication.

*Fingolimod*

Fingolimod, the first oral drug approved for the treatment of RMS, is approved for children and adult MS patients. It is a sphingosine analogue that acts by modulating the sphingosine 1-phosphate receptor (S1PR), preventing the exit of about 70% of naive B and T lymphocytes from the lymph nodes. Due to its biochemical characteristics, it can also cross the blood-brain barrier and is believed to have neuroprotective effects^42^.

Randomized Clinical Trials (FREEDOMS, TRANSFORMS, PARADIGMS) showed that Fingolimod reduces ARR by 55% compared to placebo and by 51% compared to IFN, while radiological activity was reduced by 75%^42^ ^,^ ^43^. In PARADIGMS, the reduction in ARR reached 81% compared to IFN, in line with a more inflammatory disease in younger MS patients.

Adverse effects related to Fingolimod are mild, involving upper airway symptoms, headache, paraesthesia, diarrhea, nausea and herpes zoster infection. Approximately 10% of patients experience serious adverse events^42^. Clinical and laboratorial surveillance includes transaminase elevations, lymphopenia, macular edema, and cardiovascular disorders such as bradycardia and hypertension^44^ ^,^ ^45^. Cardiac conduction blocks are more common at the beginning of medication and can be serious in a minority of cases^44^. Skin neoplasms, mainly basal cell carcinoma, were also more likely to be present^42^. These effects were also present in follow-up studies^46^.

Before starting fingolimod, it is suggested to have the following: ​​blood count, hepatic transaminases and bilirubins; varicella zoster serology OR vaccination if antibody negative; electrocardiogram; and ophthalmologic evaluation^47^. During fingolimod treatment, patients should be monitored with complete blood count, liver transaminases and bilirubins, ophthalmologic evaluation three to four months after onset and dermatologic evaluation for basal cell carcinoma^47^.

The dose of oral fingolimod is 0.5mg once daily. The first dose and doses following therapy interruption longer than 14 days should be taken in a monitored environment over six hours, with blood pressure (BP) and electrocardiogram (EKG) measurements performed before administration, assessment of BP and heart rate every hour and a new EKG at the end of the observation^42^ ^,^ ^43^ ^,^ ^47^.

Fingolimod should be one of the options in children with moderate to highly active disease or adults with mild relapses and moderate lesion burden. Due to mild relapse, moderate lesion burden and positive JCV, fingolimod was started in this case.

### Case description 06

A 30-year-old woman reported numbness in the right half of the abdomen five years ago without diagnosis. After five months she presented trigeminal neuralgia. At this time her brain MRI showed typical MS lesions (more than 10 lesions, with CEL in the brainstem lesion). No spinal cord lesion was found. CSF was positive for OCB. She referred to gestational planning in the following two years, and was concerned about close monitoring due to frequent travel for work. After a shared decision, it was decided to prescribe oral cladribine*.*

*Cladribine*

Cladribine is a purine nucleoside analogue, whose activated form accumulates in highly dividing cells, such as B- and T-cells lymphocytes and results in the disruption of cellular metabolism, the inhibition of DNA synthesis and repair and subsequent apoptosis^48^. It has rapid and sustained reductions in CD4+ and CD8+ cells and rapid, but strong, effects on CD19+ B cells, with relative sparing of other immune cells^49^. Lymphopenia therefore occurs mainly during the first months after medication. The effect of cladribine on the innate immune system is relatively limited; accordingly, neutropenia and pancytopenia are rare.

The efficacy and safety of oral cladribine versus placebo in RMS were assessed in one phase III study (CLARITY trial), in which the drug reduced the ARR by 55% and the three-month sustained progression of disability by 30%^49^. The efficacy of cladribine was confirmed by the assessment of several MRI outcomes, including brain atrophy^8^ ^,^ ^9^. In the two-year extension of this trial (CLARITY extension), cladribine produced a durable significant effect: ~75% of patients remained relapse-free despite receiving a placebo during the extension period^50^.

It is administered in only 1.75mg/kg body weight administered orally and divided into two weekly cycles in years 1 and 2.

Common side effects include fatigue and headache. More severe side effects include myelosuppression, opportunistic infections (varicella zoster, tuberculosis), nephrotoxicity, and possible increased risk of malignancy. Cladribine is contraindicated in patients with active malignancy and in patients who are or wish to become pregnant during the treatment course due to its teratogenic effect. Following completion of two treatment courses (maximum of 20 days of oral treatment in the first two years) and six months after the last dose of cladribine, it is possible to have a planned pregnancy, without taking any DMT^50^. Before putting the patient on Cladribine, CBC and hepatic enzymes should be obtained. Pregnancy and latent tuberculosis must be excluded. Monitoring includes CBC and hepatic enzymes monthly until six months of treatment and every six months afterwards^50^. 

 Due to the high efficacy and this window of opportunity for pregnancy planning, the shared decision was to start cladribine. 

### Case Description 07

A 27-year-old female patient was admitted to the neurology ward due to weakness in the right lower limb that had started about four days earlier with progressive worsening. On neurologic examination, she had grade III right crural monoparesis and a relative pupillary defect in the right-side. She reports that two years earlier she had had diplopia, lasting 20 days with spontaneous improvement. MRI showed high lesion burden, with spinal e contrast enhanced lesions. OCBs were present in CSF. She had incomplete recovery after relapse treatment. A negative anti-JCV antibody test was performed. Natalizumab was started.

*Natalizumab*

Natalizumab was the first monoclonal antibody approved for the treatment of MS. It binds to the α4 subunit of integrins (mainly α4-β1) expressed in lymphocytes, preventing their binding to vascular cell adhesion molecule 1 (VCAM-1) and the consequent migration to the central nervous system. It keeps leukocytes on the periphery, preventing action on the central nervous system^51^ ^,^ ^52^.

The main clinical trials demonstrating the benefit of Natalizumab for the treatment of MS are AFFIRM and SENTINEL. AFFIRM compared Natalizumab to placebo, demonstrating an approximate 68% reduction in AAR, in addition to an 83% and 92% reduction in T2 lesions and CEL, respectively^51^. SENTINEL compared natalizumab plus IFNβ-1a versus IFNβ-1a alone. It showed a reduction of about 54% in the annualized relapse rate, in addition to an 83% and 89% reduction in T2 lesions and contrast- enhancement, respectively^52^.

Natalizumab should be administered in an infusion center, every 28 days, within an hour, at a dose of 300mg, intravenously, with treatment options for allergic reactions easily available^51^.

Most adverse reactions are mild and usually related to the infusion. Most common are fatigue, mild allergic reactions, headache, pruritus, urinary tract and upper airway infection^51^ ^,^ ^52^. The most serious complication of the disease is related to an increased risk of developing (PML). The first reports appeared in the SENTINEL study, with two events during the follow-up of the study^52^. After these, several other cases were reported throughout the world. The main related risk factors are: time of exposure to Natalizumab, JCV antibody status and previous use of immunosuppressants. Monitoring risk factors for PML reduced the incidence of infection over the years^53^.

Before starting Natalizumab, it is suggested to perform the anti-JCV test and a baseline brain resonance within a maximum of three months before initiation. Monitoring should be performed with an anti-JCV test regularly, usually each six months and brain MRI every sixto 12 months^54^.

Natalizumab is indicated for patients with highly active MS, especially with negative anti-JCV. Due to severe relapse, high lesion burden and negative anti-JCV, Natalizumab was started for that patient. 

### Case description 08

A 39-year-old female admitted to the neurology ward due to a new paraparesis grade 2 and bladder retention since the previous week. She reported a previous episode five months earlier of a left-sided incoordination associated with ipsilateral trigeminal neuralgia, with only partial recovery, which prevented her from returning to work. She was undergoing an outpatient investigation when she presented current symptoms. MRI showed multiple periventricular, juxtacortical, middle cerebellar peduncle lesions and four eccentric short spinal cord lesions, three of them with CEL. OCBs were present in CSF and the JCV index was 1.69. She started alemtuzumab therapy after discharge.

*Alemtuzumab*

Alemtuzumab is a humanized monoclonal antibody directed against lymphocytes surface protein CD52. It targets the surface of lymphocytes and monocytes determining a deep depletion of T and B lymphocytes^55^ and leading to quantitative and qualitative changes in immune regulatory networks. These changes include suppression of memory B cells, inducing a relative increase in Treg and memory T-cell counts, and a potential shift from a pro- to anti-inflammatory environment (driven by differential reconstitution of T-cell subsets)^56^.

The CARE MS I randomized trial for highly active MS in naive patients demonstrated an annualized reduction in relapse rate of 55%, compared with interferon Beta 1a, with 77% of relapse-free patients at two-year follow-up^57^. Surprising results were found in follow-up studies, with a rate of 62% of patients remaining on NEDA 3 after five years of the initial course of alemtuzumab^58^.

Treatment with alemtuzumab occurs in two courses: in month 1 of treatment, a daily infusion of 12mg of alemtuzumab for five days and after 12 months, a daily infusion of 12mg of alemtuzumab for three days. 

Adverse effects include infusion-associated or non reactions, with up to 14% being a serious adverse effect. Up to 90% of patients report some infusion reaction, most of them with mild manifestations such as fever, headache, rash, chills and urticaria. Serious infusion reactions occurred in less than 3% of patients in the CARE MSI study^59^.

Adverse reactions not related to infusion include lymphopenia, serious infections, idiopathic thrombocytopenic purpura, thyroid disorders and kidney problems^59^ ^,^ ^60^. Because of the long-lasting effects of the drug, very close monitoring should take place for surveillance and intervention for possible harm. This includes regular assessment of thyroid and renal function with proteinuria screening and infectious monitoring. Patients should have their tuberculosis status assessed before starting treatment, receive HPV vaccine and undergo nutritional counseling to prevent Listeria foodborne infection. Due to the risk of generalized herpetic infection, prophylaxis with acyclovir is routinely given for at least one month after infusion. Also, for the prevention of infusion reactions, premedication with methylprednisolone, antipyretic and antihistamine is routinely performed in all infusions^59^ ^,^ ^61^.

In the above case, alemtuzumab was chosen as therapy in the context of highly active MS in a patient with a high JCV index, a relative contraindication to the use of natalizumab**.**

### Case description 09

A 26-year-old woman otherwise healthy presented numbness and weakness in left extremities for two weeks, with complete recovery, without a conclusive diagnosis. After six months she presented with a typical left optic neuritis. Brain MRI showed four typical brain lesions and the spinal cord MRI, a total of seven short lesions (2 CEL). OCBs were present in CSF and JCV index was 1.36. She was treated with intravenous methylprednisolone, with complete improvement. Diagnosis of RMS was done.

*Ocrelizumab*

Ocrelizumab is a recombinant humanized anti-CD20 monoclonal antibody approved for the treatment of RMS or PPMS patients^62^. It is considered a high-efficacy DMT in reducing disease activity in RMS population^63^, and moderately slowing down the progression in PPMS^64^. This treatment reduced the ARR by 45% and reduced disability progression by 40% compared with subcutaneous IFNβ-1a^63^. Analysis of brain volume loss and other MRI outcome measures also favored ocrelizumab treatment. In these trials, ocrelizumab was not associated with an increased risk of serious infections.

This therapy is initiated with a 300mg infusion, followed by a second 300mg infusion two weeks later, and subsequent 600mg dosing every six months thereafter

Common infusion-related reactions included pruritus, rash, flushing and throat irritation^63^ ^,^ ^64^. Such reactions were predominantly mild to moderate in severity and most frequently observed with the first infusion, decreasing in frequency thereafter. Opportunistic infection is reported and it is one of the therapies associated with severe COVID in MS patients^10^. Checks should be made as to immunological status, mainly HBV status and immunoglobulins level.

Due to the presence of bad prognosis factors (motor symptoms, two relapses in the first year, high spinal cord injury burden, OCB) it was proposed to initiate HET. After checking positive JCV (index 1.36), and due to not planning pregnancy, the patient started ocrelizumab.

### Case description 10

A 28-year man presented at outpatient with two episodes of limb weakness and sensitive ataxia, six months apart, in the previous year. He had been treated with intravenous steroids with incomplete recovery. MRI disclosed more than 20 typical MS brain lesions and four cervical and two thoracic CEL. OCB was positive in CSF. Positive serology for the JC virus, index 1.5 (PML risk) was found. He had a previous diagnosis of ankylosing spondylitis, with severe symptoms, only responsive to secukinumab. Anti-CD20 therapy was chosen due to possible effects on rheumatologic diseases. 

*Ofatumumab*

Ofatumumab is a fully human anti-CD20 monoclonal antibody that can be self-administered by RMS patients^65^. It presents a faster post-treatment B-cell repletion, compared to Ocrelizumab and Rituximab.

In two identical phase III trials in adults with relapsing forms of MS, subcutaneous ofatumumab was more effective than oral teriflunomide in reducing the annualized relapse rate (relative reduction of >50%), as well as reducing MRI-detected lesion activity, limiting worsening of disability and reducing serum neurofilament light chain levels. Serious infections occurred in 2.5% and 1.8% of the patients in the respective groups^66^.

Ofatumumab is offered subcutaneously at 20 mg/doses, three doses in the first month, followed by monthly doses. The injections are usually well tolerated, but mild side effects may occur (myalgia, artralgia, fever). Before putting the patient on Ofatumumab, it is necessary to check immunological status, mainly HBV status and immunoglobulins levels. Lower levels of IgM had been reported^66^.

Due to severe relapse, high lesion burden and possible effects on rheumatologic diseases, it was decided to start on a highly effective drug anti CD20. When choosing between ocrelizumab and ofatumumab, given the shorter half-life of ofatumumab and the possibility of adverse events (association of antiCD20 drugs and anti IL17 drugs), it was decided to start on ofatumumab.

## DISCUSSION

**Figure 2.**
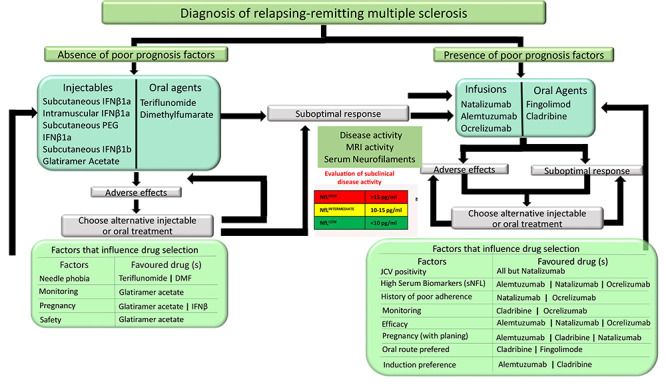
Algorithm proposal - how to choose initial therapy in MS patients.

Adapted from: D. Rotstein and X. Montalban, “Reaching an evidence-based prognosis for personalized treatment of multiple sclerosis,” Nature Reviews Neurology, vol. 15, no. 5, pp. 287-300, May 2019, doi: 10.1038/s41582-019-0170-8. tefan Bittner, Jiwon Oh, Eva Kubala Havrdová, Mar Tintoré, Frauke Zipp, The potential of serum neurofilament as biomarker for multiple sclerosis, Brain, Volume 144, Issue 10, October 2021, Pages 2954-2963.

IFN-B: beta interferon; DMF: Dimethyl-fumarate; JCV: John Cummingham vírus ; MRI: Magnetic ressonance Imaging; Nf-L: Neurofilament Light Chain levels.

Early treatment of MS is beneficial, but a definitive algorithm on how to choose the first DMT in MS patients is still lacking in current literature (Figure 2). Short-term benefit and safety among different DMT are uncertain, since few comparative long-term studies are available^67^. The risks of disability and therapeutic response of different DMTs can be estimated considering individual patient characteristics, disease onset, activity level and treatment particularities^68^.

Patients’ prognostic factors include age, gender, ethnicity, environmental factors and comorbidities. Male gender is associated with early disability and progression, and non-Caucasian ethnicity is related to delayed diagnosis and increased disability^69^. Age is a key predictor of disability. Age-related iron accumulation, a process occurring physiologically in the human brain, reaching a plateau around 50 years, is increased in MS brains. Iron-accumulation is released from damaged oligodendrocytes and myelin during active demyelination. MS pathology comprises two different stages, an initial predominantly inflammatory phase that evolves to a neurodegenerative phase. In line, younger patients have a more inflammatory disease and better treatment response. The efficacy of DMT declined markedly with increased age^70^ and treatment benefits are lower for MS patients aged >50 years. Early treatment can prevent disability worsening either associated with or independent of relapses and seems to endorse the early use of HET in patients with highly active MS. Additional work is necessary to determine the role of age, along with other disease characteristics, in individual treatment decision-making^68^.

Environmental and personal modifiable factors, such as vitamin D deficiency, smoking, sedentarism and comorbidities (Hypertension, Diabetes, Obesity, ischemic heart disease, epilepsy, and psychiatric diseases), predict earlier disability. A higher comorbidity burden has been associated with greater risks of relapse and disability progression. These conditions may impact brain function, exacerbate brain atrophy and interact with smoldering MS lesions, and may explain the more rapid worsening of disability^71^. It is reasonable that proper management of clinical comorbidities and other modifiable factors lead to a better prognosis; however, their role to stratify individual treatment decisions is not clear [68]. Inflammatory systemic biomarkers are associated with higher risk of active and progressive disease, but so far there is no robust evidence for establishing its use in clinical decisions^72^.

Besides the above, other known prognostic factors are related to disease onset and activity. Optic neuritis and sensory manifestations in the first relapse predict a better disease course than motor, sphincter, brainstem, cerebellar and cognitive dysfunctions. Moreover, a short time between initial relapses, mainly if less than two years apart, indicates a worse prognosis^69^. DA should also be better assessed to determine the best treatment option. Most drugs showed more favorable results reducing ARR in the subgroup of RMS patients with high disease activity (HDA), both relapse and MRI activity, and early HET should be recommended^68^.

MRI activity, new T2-lesions and CEL, appears to be a more sensitive measure of disease activity compared to relapses and is the most useful biomarker in clinical practice. The presence of OCB in CSF indicates a higher risk of conversion of future disability. Other systemic inflammatory biomarkers have been associated with relapse (ESR, PCR) and progression (homocysteine)^73^ ^,^ ^72^. Levels of serum neurofilaments (sNfl), a major component of neuronal and axonal cytoskeleton proteins, reflect ongoing inflammatory-driven neuroaxonal damage. sNfL levels predict recurrence, correlate with CEL , T2 lesion load and brain and spine atrophy and can be used as an additional measure of disease activity. Recently, Glial fibrillary acidic protein (GFAP), the major cytoskeleton protein in astrocytes released upon changes in cellular integrity, has increased in HAD^74^.

DMT classification according to efficacy is interesting for future comparative studies (Table 1), but an international consensus is crucial. Current knowledge of comparative DMT efficacy is usually inferential, since the ideal head-to-head trials of long duration are not available^75^. For instance, the classification of fingolimod as a HET deserves some caveats. The ongoing studies (DELIVER MS^76^ and TREAT-MS^77^), for example, classify fingolimod as MET, although its efficacy seems to be superior to DMF^78^, comparable to cladribine, but inferior to that other HET such as ocrelizumab, ofatumumab, natalizumab or alemtuzumab^79^.

Observational studies suggest that initial treatment with a HET strategy is associated with a lower risk of conversion to SPMS in patients with DA^7^ ^,^ ^12^ ^,^ ^53^ ^,^ ^63^ ^,^ ^67^. A recent study compared patients treated initially with HET to those with MET and found a decreased risk of six-month confirmed EDSS deterioration and a lower probability of on-treatment relapses^80^. Growing evidence suggests that early HET is beneficial to the disease course for most RMS patients. However, people have individual risk-taking profiles, and risk aversion is associated with older age, female sex and socio-economic status^4^. HET have been associated with serious adverse events in MS patients (Table 2) and could be associated with risks that are unknown until the post marketing phase, as was the case for PML associated with natalizumab and immune-mediated encephalitis associated with daclizumab^68^. MS is a chronic disease, and early HET exposes patients to substantial risks that increase and unknown long-term effects, such as risks of malignancy or chronic immunodepletion. Data from various international registraties studies indicate that MS patients may be at higher risk of acquiring COVID 19, of experiencing severe COVID 19 and death than the general population. Treatment with anti CD20 therapy (ocrelizumab, rituximab or ofatumumab) is a risk factor for severe COVID, while IFN and teriflunomide may be protective^10^.

**Table 2.** Safety issues and risk minimization strategies in disease-modifying therapies for relapsing multiple sclerosis.

	Adverse effects	Risk of infections‡	Monitoring	Special population
Moderate efficacy				
Interferon Beta	Headache, injection site reactions,flu-like symptoms, elevated liver enzymes, psychiatric symptoms	Not increased	CBC and liver enzymes every 6 months	Relatively safe during pregnancy and lactation. Can be used in children
Glatiramer Acetate	Injection site reactions, palpitation, dyspnea, anxiety	Not increased	Not needed	Relatively safe during pregnancy and breastfeeding
Dimethyl fumarate	Flushing, gastrointestinal symptoms, liver and renal toxicity	Not increased	CBC every 6 months	Limited data during pregnancy. Breastfeeding contraindicated
Teriflunomide	Headache, GI symptoms, hair thinning, liver enzymes increase	Not increased	Liver enzymes monthly for 6 months, then every 6 months	Potentially teratogenic. Accelerate drug elimination in case of accidental pregnancy
High efficacy				
Fingolimod Cladribine	Headache, hypertension, macular edema, liver toxicity, bradyarrhythmia Headache, lymphopenia, nausea, malignancy	May be increased for herpes virus and respiratory infections Slightly increased risk of overall infection rate and serious infections AE	CBC and liver enzymes every 6 months. Fundoscopy before and 3-4 months after start CBC 2 and 6 months after each course, cancer screening according to age	Can be used in children older than 10yo. Not recommended during pregnancy and lactation Avoid pregnancy for 6 months after the last treatment course. Breastfeeding allowed after 7 days
Ocrelizumab	Infusion reactions, headache, malignancy risk potential	Increased risk of upper respiratory, UTI and herpes virus infections	CBC and liver enzymes annually	Avoid pregnancy for at least 6-12 months after treatment
Ofatumumab	Infections, injection site reactions, headache	Not increased	CBC and liver enzymes annually	Avoid pregnancy for at least 6-12 months after treatment
Natalizumab	Fatigue, allergic reactions, headache, PML	Not increased, except for JCV-PML cases	JCV/ PML screening every 3-6m, CBC and liver enzymes every 6 months	High risk of relapse if stopped, Consider maintenance of treatment until 32-36 weeks of pregnancy. Low absorption in breastfeeding
Alemtuzumab	Infusion reactions, infections, thyroid disorder and other autoimmune conditions (ITP, kidney disease)	Increased risk of overall infection rate, and serious infections AE.	CBC, renal function, urinalysis every month and TSH every 3 months until 2 years after last cycle; cancer screening annually	Possibility of pregnancy and breastfeeding after 4 months of last infusion. Monitor development of autoimmune diseases during pregnancy.

AE: adverse events ; ARR: Annualized Relapse Rate; CBC: complete blood count ; NEDA: No Evidence of Disease Activity; INFB: Beta interferonas; ITP : immune thrombocytopenia; JCV: John Cunningham Virus; Peg INF: beta interferona peglada; PML: progressive multifocal leukoencephalopathy; †Data from pivotal trials compared to control group (either placebo or active group).

For patients with good prognostic factors, the use of MET may be a proper approach, since HET is not suitable for all patients and requires an individual risk-benefit assessment. Patients’ wishes and risk-taking profile should be valued in a shared decision- making^68^. Applying personalized predictive models to MS patients is a new field that is rapidly evolving, including artificial intelligence methods.

**Figure 3.**
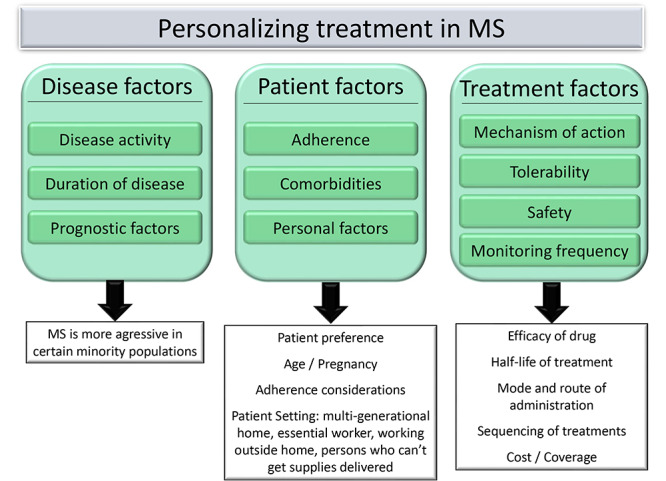
A personalized approach.

A personalized approach may guide towards the best shared treatment decision between patient and physician (Figure 3). For all patients starting on a DMT, it is advisable to perform a complete laboratory analysis, vaccination status check, and consider the infection risk, which will also help on the treatment choice^81^. When starting or switching treatment, it is essential to continuously monitor patients and assess treatment response, including a thorough neurological examination, laboratory and neuroimaging at regular intervals^10^ ^,^ ^81^.

This paper provides practical information on how to manage the challenge of choosing first-DMT based on real-life cases, with a critical view of current scientific evidence. However, it has a few limitations. We did not discuss autologous hematopoietic stem cell transplantation or mitoxantrone, since these treatment modalities are rarely used in clinical practice nowadays. The most usual scheme of categorize DMT according to efficacy is ARR reduction, then it was used in this paper. Though, other criteria, such as PIRA reduction, may be more useful in prevent disability . Some DMTs like INF and GA presents around 30% in ARR reduction and were classified as MET herein, as usually is reported in literature. Even so, we believe that a classification in low, moderate and high efficacy therapy may be more appropriate. Also, we did not perform a systematic review, since we aimed to only evaluate medications approved in Brazil, with a practical and critical review concerning those treatments.

In conclusion, advances in MS treatment are remarkable. Strong evidence supports the use of early HET. However, biomarkers, clinical and radiologic prognostic factors, as well as patients’ individual issues, should be valued and considered for a personalized treatment decision.
